# Early social isolation differentially affects the glucocorticoid receptor system and alcohol-seeking behavior in male and female Marchigian Sardinian alcohol-preferring rats

**DOI:** 10.1016/j.ynstr.2023.100598

**Published:** 2023-12-07

**Authors:** F. Benvenuti, S. De Carlo, L. Rullo, L. Caffino, L.M. Losapio, C. Morosini, M. Ubaldi, L. Soverchia, N. Cannella, E. Domi, S. Candeletti, F. Mottarlini, L. Fattore, P. Romualdi, F. Fumagalli, V. Trezza, M. Roberto, R. Ciccocioppo

**Affiliations:** a School of Pharmacy, Center for Neuroscience, Pharmacology Unit, University of Camerino, Camerino, Italy; b Department of Pharmacy and Biotechnology, Alma Mater Studiorum-University of Bologna, Bologna, Italy; c Department of Pharmacological and Biomolecular Sciences, ‘Rodolfo Paoletti’, University of Milan, Milan, Italy; d CNR Institute of Neuroscience-Cagliari, National Research Council, Cagliari, Italy; e Department of Science, University “Roma Tre”, Rome, Italy; f Department of Molecular Medicine, The Scripps Research Institute, La Jolla, CA, USA

**Keywords:** Alcoholism, Reward, Glucocorticoid receptor, Alcohol drinking, Stress, Relapse

## Abstract

Adverse early life experiences during postnatal development can evoke long-lasting neurobiological changes in stress systems, thereby affecting subsequent behaviors including propensity to develop alcohol use disorder. Here, we exposed genetically selected male and female Marchigian Sardinian alcohol-preferring (msP) and Wistar rats to mild, repeated social deprivation from postnatal day 14 (PND14) to PND21 and investigated the effect of the early social isolation (ESI) on the glucocorticoid receptor (GR) system and on the propensity to drink and seek alcohol in adulthood. We found that ESI resulted in higher levels of GR gene and protein expression in the prefrontal cortex (PFC) in male but not female msP rats. In female Wistars, ESI resulted in significant downregulation of *Nr3c1* mRNA levels and lower GR protein levels. In male and female msP rats, plasma corticosterone levels on PND35 were similar and unaffected by ESI. Wistar females exhibited higher levels of corticosterone compared with males, independently from ESI. In alcohol self-administration experiments we found that the pharmacological stressor yohimbine (0.0, 0.312, 0.625, and 1.25 mg/kg) increased alcohol self-administration in both rat lines, regardless of ESI. After extinction, 0.625 mg/kg yohimbine significantly reinstated alcohol seeking in female rats only. ESI enhanced reinstatement in female msP rats. Overall, the present results indicate that repeated social deprivation during the third week of postnatal life affects GR expression in a strain- and sex-dependent manner: such effect may contribute, at least partially, to the heightened sensitivity of female msP rats to the effects of yohimbine-induced alcohol seeking.

## 1 Introduction

Alcohol dependence is a multifactorial disorder in which genetic and environmental factors interact to determine an individual's vulnerability or resilience to developing it (Ducci and Goldman, 2008; Enoch, 2011; Young-Wolff et al., 2011). Clinical and preclinical studies suggest that adverse social experiences during early stages of postnatal life are associated with alterations of synaptic and neuronal development that negatively affect proper development of the brain (Chatterjee et al., 2007; Marco et al.; Sale et al., 2014), leading to cognitive, emotional, and social impairment (Hassel et al., 2011) and a greater susceptibility to psychiatric disorders (Turecki et al., 2014), including alcohol use disorder and other substance use disorders (Brenhouse et al., 2013; Nylander and Roman, 2013). Indeed, exposure to maltreatment and cumulative stressful life events before puberty, particularly in the first few years of life, is associated with an early onset of problem drinking in adolescence and alcohol use disorder and substance use disorders in adulthood (De Bellis, 2002; Hyman et al., 2006; Nelson et al., 2006; Pilowsky et al., 2009). Rodent studies showed that exposure to stress during early postnatal life modulates the rewarding effects of cocaine, amphetamine, and morphine in adulthood (Moffett et al., 2006; Francis and Kuhar, 2008; Boasen et al., 2009; Lewis et al., 2013). A higher propensity for alcohol drinking was also demonstrated (Roman and Nylander, 2005; Moffett et al., 2007; Nylander and Roman, 2013; Palm et al., 2013; García-Gutiérrez et al., 2016; De Almeida Magalhães et al., 2017; Odeon et al., 2017; Amancio-Belmont et al., 2020).

One key system that mediates the stress response is the hypothalamic-pituitary-adrenal (HPA) axis, and stress exposure in early life has been shown to significantly impact HPA axis function increasing susceptibility to psychopathology later in life (Van Bodegom et al., 2017; Liu and Nusslock, 2018; Nishi, 2020). Despite some inconsistent reports, there is consensus that stress exposure in early life results in HPA axis hyperactivity in adulthood, with increases in corticotropin-releasing factor signaling and impairments in glucocorticoid receptor (GR)-mediated negative feedback (Van Bodegom et al., 2017). Preclinical models that attempt to mimic early traumatic events in rodents have primarily focused on the first 2 weeks of life. However, recent studies have shown that during the juvenile period in rodents (postnatal weeks 3–4), a large-scale reconfiguration of the neuronal epigenome and extensive synaptogenesis occur, similar with what occurs in humans during childhood (Lister et al., 2013). In rodents, this developmental time-window is also characterized by the maturation of visual, motor, and social functions that are crucial for interactions with the environment (Pellis and Pasztor, 1999; Berardi et al., 2000; Rice and Barone, 2000). Notably, early social isolation (ESI) during the third postnatal week in mice increased cocaine-induced conditioning place preference compared with controls (Lo Iacono et al., 2016, 2017). Moreover, it has been demonstrated that mice exposed to the ESI protocol (i.e., single housing in a cage with clean bedding for 30 min/day from PND 14 to PND 21), which we decided to use for the present study, exhibited depressive-like behaviors in adulthood associated with epigenetic changes in different brain regions (Catale et al., 2020; Lo Iacono et al., 2015). Thus, in the present study, we decided to use this same ESI protocol that did not affect locomotor activity in adolescent rat offspring (Bratzu et al., 2023), suggesting that it induces subtle adaptations without disrupting general animal behavior. Although there has been a greater perception of the importance of social isolation during early adolescence for the subsequent development of psychiatric disorders, very little has been investigated with regard to problematic drinking.

In the present study, we hypothesized that exposure to mild, repeated social isolation during the third postnatal week induces stable molecular changes at the level of the GR system that likely alter responses to alcohol reward and increases the susceptibility to alcohol seeking. To this end, we applied the ESI protocol and explored the long-term consequences of this adverse early social experience on HPA axis evaluating plasmatic corticosterone (CORT) levels and GR expression and trafficking in the amygdala (Amy) and prefrontal cortex (PFC), two brain regions that are critically linked to stress and alcohol drinking in rodents. ESI-exposed rats were subsequently tested for their vulnerability to develop excessive drinking and seeking. To assess whether environmental stress interacts with heritable factors, we used unselected Wistar rats and genetically selected Marchigian Sardinian alcohol-preferring (msP) rats, a rodent line that is characterized by heightened alcohol consumption and stress sensitivity (Ciccocioppo et al., 2006; Borruto et al., 2021). The majority of studies have focused on male rats only; very few reports have examined the consequences of early life stress in females (Lundberg et al., 2017). Clinical evidence supports a higher association between a history of child maltreatment and a higher risk to develop drug use and psychiatric disorders in women than in men (Dinwiddie et al., 2000; Anda et al., 2002; Osofsky et al., 2021). Women initiate alcohol consumption more frequently than men as a coping strategy to attenuate negative affective states, such as anxiety, depression, and posttraumatic stress disorder (Crutzen et al., 2013; Peltier et al., 2019; Guinle and Sinha, 2020). Women are also more likely to relapse in response to stressful events (Greenfield et al., 2007; Hyman et al., 2008; Hudson and Stamp, 2011). The vulnerability to several psychopathological conditions is also well known to display sex differences in experimental animals (Zagni et al., 2016; Palanza and Parmigiani, 2017), with notable sex differences in HPA axis function (Heck and Handa, 2019; Peltier et al., 2019). Therefore, in the present study, we used both male and female msP and Wistar rats to investigate the effect of ESI on the GR system and its impact on the propensity to drink and seek alcohol in adulthood. Additionally, we assessed the ability of yohimbine, a pharmacological stressor, to increase alcohol consumption and relapse to alcohol seeking. Yohimbine was chosen because earlier studies demonstrated that it induces anxiety-like responses and enhances motivation for alcohol in both humans and rodents, providing evidence of the translational value of this approach (Curley et al., 2022; Bremner et al., 1996b; Holmberg and Gershon, 1961; Umhau et al., 2011).

## 2 Materials and methods

### 2.1 Animals

Male (*n* = 28/line) and female (*n* = 25–27/line) msP and Wistar rats were used in the study. All rats were bred at the animal facility of the University of Camerino, Italy. msP and Wistar dams were singly mated with an individual male rat of the same line until pregnancy could be verified. At this point, the male rat was removed from the cage, and pregnant females were single housed until delivery. The breeding colony was kept on a 12 h/12 h light/dark cycle (light on at 7 a.m.). All animals were disturbed as little as possible during the breeding process and had *ad libitum* access to food pellets (4RF18, Mucedola, Settimo Milanese, Italy) and tap water. The day of birth was considered postnatal day 0 (PND0). At birth, the litters were left undisturbed with their mothers until PND14, at which time half of the pups from each nest was subjected to the ESI protocol as described below. On PND21, all pups were weaned and housed in groups of the same sex and environmental condition in a new room on a 12 h/12 h light/dark cycle (light off at 8 a.m.). They were housed under same-sex/environmental condition groups of 3 or 4 per cage with *ad libitum* access to food and water. All subsequent experiments began on PND35, at which time they weighed approximately 150 g (males) and 130 g (females). Before starting the behavioral experiments, the rats were handled daily for 5 min for 3 days by the same researchers who performed the experiments. The experiments were conducted during the dark phase of the light/dark cycle.

All efforts were made to minimize animal suffering and reduce the number of animals used. All animal procedures were conducted in adherence to the European Community Council Directive for Care and Use of Laboratory Animals and the National Institutes of Health Guide for the Care and Use of Laboratory Animals. The animal studies are reported in compliance with the ARRIVE 2.0 guidelines (Percie du Sert et al., 2020).

### 2.2 Early social isolation protocol

Early social isolation occurred during the light cycle, between 9:00 and 10:00 during PND14 - PND21, as previously described (Bratzu et al., 2023; Catale et al., 2020; Lo Iacono et al., 2016). Half of the pups from each nest were singly housed in a novel clean bedding cage for 30 min per day (i.e., each pup was isolated from both the dam and the siblings). After the 30 min separation time, they were returned to their home cages. Control pups were left undisturbed with their mothers in their home cages. Half of the pups from each nest were individually removed and placed in a cage with clean bedding for 30 min/day. After the 30 min separation time, they were returned to their home cages. Control pups were left undisturbed with their mothers in their home cages.

### 2.3 Tissue collection

On PND35, the animals were sacrificed under low stress conditions. They were deeply anesthetized with isoflurane and quickly decapitated. Blood was collected from the trunk, and brain areas of interest were dissected for *in vitro* experiments and quickly frozen on dry ice. Dissection was carried out using a rat brain matrix (Stoelting, USA) using a classical protocol described by Heffner et al. (1980). Briefly, the brains, quickly removed from the skull, were placed on the moulding block, and with the help of a blade, they were cut to obtain coronal sections. At this point, the portion of the prefrontal cortex was taken as is, while the amygdala was isolated using a puncher. Tissues were stored at −80 °C until the gene and protein expression analyses were performed.

### 2.4 RNA extraction and gene expression analysis by quantitative real-time polymerase chain reaction

Total RNA was extracted according to the method of Chomczynski and Sacchi (Chomczynski and Sacchi, 1987). Each sample was subjected to DNase treatment and converted to cDNA with the GeneAmp RNA PCR kit (Life Technologies) as previously described (Caputi et al., 2021). Quantitative real-time polymerase chain reaction (qRT-PCR) was performed with a StepOne Real-Time PCR System (Life Technologies) using SYBR Green PCR MasterMix (Life Technologies). The relative expression of different gene transcripts was calculated using the ΔΔCt method and converted to relative expression (2^−ΔΔCt^) for the statistical analysis (Livak and Schmittgen, 2001). All data were normalized to the housekeeping gene glyceraldehyde-3-phosphate dehydrogenase (GAPDH). The specificity of each PCR product was determined by melting curve analysis, constructed in the range of 60 °C–95 °C. The primer sequences that were used for PCR amplification were designed using Primer 3 and were the following: *Gapdh* (forward, 5′-AGACAGCCGCATCTTCTTGT-3’; reverse, 5′-CTTGCCGTGGGTAGAGTCAT-3′), *Nr3c1* gene encoding for GR (forward, 5′-GAAAAGCCATCGTCAAAAGGG-3’; reverse, 5′-TGGAAGCAGTAGGTAAGGAGA-3′).

### 2.5 Preparation of protein extracts and western blot analyses

PFC tissue was homogenized in a glass-glass potter using cold buffer that contained 0.32 M sucrose, 1 mM HEPES solution, 0.1 mM ethylene glycol tetra-acetic acid (EGTA), and 0.1 mM phenylmethylsulfonyl fluoride, pH 7.4, in the presence of a complete set of protease inhibitors and a phosphatase inhibitor cocktail. The homogenized tissues were centrifuged at 1000 ⋅ *g* for 10 min. The resulting pellet (P1), corresponding to the nuclear fraction, was resuspended in a buffer that contained 20 mM HEPES, 0.1 mM dithiothreitol, and 0.1 mM EGTA, with protease and phosphatase inhibitors. The supernatant (S1) was centrifuged at 9000 ⋅ *g* for 15 min to obtain the pellet that corresponded to the crude synaptosomal fraction, and the resulting supernatant S2 corresponded to the clarified fraction of cytosolic proteins. Total proteins were measured in the nuclear fraction and cytosolic fraction using the Bio-Rad Protein Assay, with bovine serum albumin as the calibration standard (Bio-Rad Laboratories, Segrate, Milan, Italy). GR levels were evaluated in both the nuclear and cytosolic fractions. Ten micrograms of proteins for each sample were run on sodium dodecyl sulfate-10% polyacrylamide gel under reducing conditions and then electrophoretically transferred to nitrocellulose membranes (Bio-Rad Laboratories). Blots were blocked 1 h at room temperature with 10% bovine serum albumin in TBS buffer and then incubated with the anti-GR antibody (1:500, Thermo Scientific, USA). The results were standardized using β-actin (1:10,000, Sigma-Aldrich, Milan, Italy) as the control protein, which was detected by evaluating the band density at 43 kDa. Immunocomplexes were visualized by chemiluminescence using the Chemidoc MP Imaging System (Bio-Rad Laboratories) and analyzed using Image Lab software (Bio-Rad). Gels were run twice each. The results represent the average from two different Western blots and were averaged and normalized using a specific correction factor (Caffino et al., 2020). Examples of full-size original cropped immunoblots of protein expression levels that were measured in the nuclear and cytosolic fractions of the PFC are presented in Supplementary Figs. S3–4).

### 2.6 Analysis of plasma corticosterone levels

Blood samples from each rat were collected in tubes that contained ethylenediaminetetraacetic acid (EDTA; 250 μl ⋅ 2 ml of collected blood) as the anticoagulant agent. Plasma was separated by centrifugation at 6500 ⋅ *g* for 10 min. Corticosterone (CORT) levels were determined using an enzyme-linked immunosorbent assay (ELISA) with a commercial kit according to the manufacturer's instructions (Tecan, Italy).

### 2.7 Drugs

The alcohol drinking solution 10% (v/v) was prepared by diluting 95% alcohol (F.L. Carsetti, Camerino, Italy) with tap water. Yohimbine hydrochloride (17-hydroxyyohimban-16-carboxylic acid methyl ester hydrochloride) was purchased from Sigma-Aldrich, Italy, dissolved in sterile distilled water, and administered intraperitoneally (i.p.) at 0.0, 0.312, 0.625, and 1.25 mg/kg in a 1 ml/kg injection volume 30 min before the drug tests (Lê et al., 2005; Marinelli et al., 2007; Ayanwuyi et al., 2013).

### 2.8 Self-administration apparatus

Operant alcohol self-administration training and drug testing were conducted in standard operant conditioning chambers (Med Associates, St. Albans, VT, USA) that were enclosed in ventilated sound-attenuating cubicles. Each chamber was equipped with two retractable levers in the front panel that were positioned laterally to the drinking reservoir and connected to a syringe pump. A house light was located on the wall opposite the levers. A Windows-compatible computer with Med-PC-5 software (Med Associates) controlled the delivery of fluid, presentation of visual stimuli, and recording of behavioral data.

### 2.9 Experiments

EXPERIMENT 1: *Effect of ESI on GR gene and protein levels and plasma CORT levels.* Twelve male (*n =* 6/environmental condition) and 12 female (*n =* 6/environmental condition) msP rats and 12 male (*n =* 6/environmental condition) and 12 female (*n =* 6/environmental condition) Wistar rats were sacrificed at the beginning of the dark cycle on PND35. This time point was chosen since late adolescence is an age of extreme vulnerability to the development of psychiatric disorders, thus interfering with the trajectory of maturation might lead to aberrant reward processing (Sale et al., 2014). Blood samples were collected to measure CORT levels. The transcriptional levels of the gene encoding for GR, *Nr3c1*, were evaluated in the PFC and Amy, whereas GR protein levels were investigated in the PFC.

*EXPERIMENT 2: Effect of ESI on alcohol self-administration.* To examine whether ESI-induced molecular changes at the level of the GR system affect the response to alcohol, alcohol-related behaviors were evaluated in a new cohort of rats. Behavioral training began on PND35. Sixteen male (*n* = 8/environmental condition) and 15 female (*n* = 8–7/environmental condition) msP rats and 16 male (*n* = 8/environmental condition) and 13 female (*n* = 6–7/environmental condition) Wistar rats were used in this experiment. On PND35, the rats were given intermittent access to 10% (v/v) alcohol in an additional water bottle in their home cage for 1 week. The purpose of this procedure was to avoid neophobic responses to alcohol in the operant chambers. After this acclimation period, operant training began. The rats were given 15 h access to a single lever (right lever) that produced 0.1 ml deliveries of water on a fixed-ratio 1 (FR1) schedule of reinforcement with *ad libitum* food available on the floor of the operant chamber. Afterward, the animals were trained to respond for 10% (v/v) alcohol in 30 min daily sessions under a FR1 schedule of reinforcement. Operant sessions began with lever extension into the chamber and ended with lever retraction. Responses at the right (active) lever were reinforced with 0.1 ml of 10% (v/v) alcohol that was delivered in the drinking reservoir. Reinforcement delivery was followed by a 5 s timeout (TO) period, during which the house light was contingently illuminated. During the TO, active lever responses were recorded but not reinforced. Throughout the sessions, responses at the left (inactive) lever had no scheduled consequences. The number of operant responses at both the active and inactive levers and the number of reinforcers received were recorded. Alcohol self-administration training was performed 5 days weekly until the successful acquisition of a stable baseline of operant responding was achieved (18 sessions).

*EXPERIMENT 3: Effect of ESI on alcohol self-administration on a progressive-ratio schedule of reinforcement.* After the successful acquisition of operant responding under the FR1 schedule of reinforcement, the same cohort of animals in the previous experiment was switched to a progressive-ratio (PR) schedule of reinforcement to evaluate their motivation for alcohol (Ayanwuyi et al., 2013; Kallupi et al., 2017). The PR schedule is an operant schedule that measures the maximum amount of work an animal is willing to expend to obtain a reward, reflecting its motivation for it (Richardson and Roberts, 1996). The breakpoint, defined as the last ratio completed by the animals to obtain one dose of 10% (v/v) alcohol, was used as a measure of motivation. Under the PR contingency, the response requirement that was necessary to receive one dose of 10% (v/v) alcohol was increased according to the following progression: 1, 2, 3, 4, 6, 8, 10, 12, 16, 20, 24, 28, 32, 36, 40, 44, 48, 52, 56, 60, 64, 68, 72, 76, 80, 84, 88, 92, 96, 100, 104. Each alcohol-reinforced response resulted in the house light being turned on for 5 s, and sessions were terminated when more than 30 min elapsed since the last reinforced response.

*EXPERIMENT 4: Effect of ESI on alcohol self-administration following yohimbine administration.* Following the PR test, the animals were returned to an FR1 schedule of reinforcement to restore the alcohol self-administration baseline. Once stable self-administration responding was obtained (in 5 days) under this reinforcement schedule, the experiment started. The pharmacological stressor yohimbine was used at doses (0.312, 0.625, and 1.25 mg/kg) that were previously shown to increase alcohol-reinforced lever pressing in both Wistar and msP rats (Marinelli et al., 2007; Ayanwuyi et al., 2013). To habituate animals to the injection procedure, physiological saline was injected i.p. Three times before drug testing. Yohimbine (0.312, 0.625, 1.25 mg/kg) or its vehicle was administered i.p. 30 min before the self-administration session using a within-subjects counterbalanced Latin-square design. Drug tests were conducted every fourth day. Following each test day, the animals were allowed one day off, and a new baseline was then established over the next 2 days. The number of operant responses at both the active and inactive levers and number of reinforcers received were recorded.

*EXPERIMENT 5: Effect of ESI on yohimbine-induced reinstatement of alcohol seeking.* The experimental procedure consisted of three phases: operant training, extinction, and reinstatement. Briefly, the same cohort of animals that was used in the previous experiments was retrained for FR1 alcohol self-administration for 10 days to reestablish a stable baseline of operant responding. The rats were then subjected to 16 daily 30 min extinction sessions, during which lever presses were no longer associated with alcohol delivery, but the house light was still presented to allow for its concomitant extinction. At completion of the extinction phase, the rats were challenged with a single dose (0.625 mg/kg) of yohimbine, and reinstatement was evaluated. Because in the previous experiment, 0.625 mg/kg yohimbine was sufficient to increase alcohol self-administration in all groups, this dose was again used in the reinstatement experiment. Additionally, previous studies demonstrated that this dose of yohimbine can reinstate alcohol seeking in both msP and Wistar rats (Ayanwuyi et al., 2013). Yohimbine (0.625 mg/kg) was administered i.p. 30 min before the 30 min reinstatement session that was conducted under identical conditions to extinction training. Total responses at the active lever were recorded and used to evaluate alcohol-seeking behavior. Inactive lever responses were also measured.

### 2.10 Statistical analysis

Data from msP and Wistar rats were analyzed independently for each experiment. Biochemical and molecular data have been initially evaluated by Shapiro-Wilk test to confirm the normality of the distribution and by Grubb's or Dixon's Q (for small data sets) test to identify outliers. Identified outliers were excluded from statistical analysis. They are indicated in figure legends and are depicted in the figures of the Supplementary Material (Figures S1, S2 and S5). After testing for the assumption of a normal distribution, changes in mRNA, proteins and plasma CORT levels were analyzed using two-way analysis of variance (ANOVA), with ESI and sex as between-subjects factors. When appropriate, the Newman-Keuls test was used for *post hoc* comparisons. To evaluate the operant self-administration training data, the number of alcohol rewards was analyzed using three-way ANOVA, with ESI and sex as between-subjects factors and sessions as the repeated measure. The breakpoint for alcohol self-administration under the PR schedule of reinforcement was evaluated using two-way ANOVA, with sex and ESI as between-subjects factors. The effect of yohimbine on alcohol self-administration was analyzed using three-way ANOVA, with sex and ESI as between-subjects factors and treatment as the repeated measure. Active and inactive lever responses were analyzed separately. The yohimbine-induced reinstatement results were analyzed using three-way ANOVA, with sex and ESI as the between-subjects factor and reinstatement as the repeated measure. When appropriate, the Newman-Keuls test was used for *post hoc* comparisons. Values of *p* < 0.05 were considered statistically significant.

## 3 Results

### 3.1 EXPERIMENT 1: effect of ESI on Nr3c1 mRNA levels, GR protein levels and plasma CORT levels

**Fig. 1:**
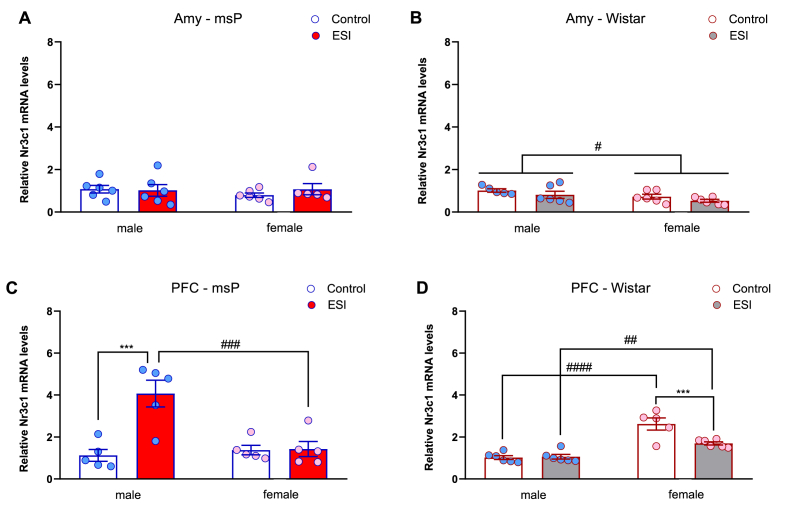
**Effect of early social isolation on *Nr3c1* mRNA levels in the Amy and PFC of male and female msP and Wistar rats.** (**A**, **B**) *Nr3c1* mRNA levels in the Amy in male and female msP (N = 5–6/group) and Wistar rats (N = 6/group; 1 outlier in male control group). (**C**, **D**) *Nr3c1* mRNA levels in the PFC in male and female msP (N = 5–6/group; 1 outlier in male and female control group, 1 outlier in female ESI group) and Wistar rats (N = 6/group; 1 outlier in female control group). The data represent 2^−ΔΔCt^ values calculated using the ΔΔCt method and were normalized to *Gapdh* as the housekeeping gene. The data are expressed as the mean ± SEM. Male msP ESI *vs* female msP ESI (PFC): ^###^*p <* 0.001; male msP control *vs* male msP ESI (PFC): ***p < 0.001; main effect of sex (Wistar Amy): ^#^; male Wistar control *vs* female Wistar control: ^####^*p <* 0.001; male Wistar ESI *vs* female Wistar ESI: ^##^*p <* 0.01; female Wistar control *vs* female Wistar ESI: ***p < 0.001; Where not indicated, differences from controls were not statistically significant.

We tested the effect of ESI on *Nr3c1* mRNA levels in the Amy and PFC of male and female msP and Wistar rats on PND35. In the Amy of msP rats, two-way ANOVA revealed no significant effect of sex (*F*_1,19_ = 0.29, *p* = 0.59) or ESI (*F*_1,19_ = 0.28, *p* = 0.60) and no sex × ESI interaction (*F*_1,19_ = 0.64, *p* = 0.44; Fig. 1A). In Wistar rats, the overall ANOVA showed a main effect of sex (*F*_1,19_ = 5.99, *p* = 0.02), but no significant effect of ESI (*F*_1,19_ = 2.83, *p* > 0.05) or sex × ESI interaction (*F*_1,19_ = 0.002, *p* = 0.95; Fig. 1B). Two-way ANOVA of *Nr3c1* mRNA levels in the PFC of msP rats revealed significant effects of sex (*F*_1,16_ = 8.80, *p* = 0.01) and ESI (*F*_1,16_ = 13.41, *p* = 0.002) and a sex × ESI interaction (*F*_1,16_ = 12.53, *p* = 0.027). The *post hoc* analysis showed that male msP rats subjected to ESI during the third week of postnatal life exhibited significantly higher *Nr3c1* mRNA levels compared to msP male controls (*p* < 0.001). A significant difference between the male msP ESI and female msP ESI groups were also detected (*p* < 0.001). No significant difference was found for *Nr3c1* mRNA levels in female msP rats subjected to ESI compared with the respective control group (*p* > 0.05; Fig. 1C). In the PFC of Wistar rats, two-way ANOVA revealed a significant effect of sex (*F*_1,19_ = 54.82, *p* < 0.0001), ESI (*F*_1,19_ = 8.56, *p* = 0.008) and a sex × ESI interaction (*F*_1,19_ = 10.16, *p* = 0.004). The *post hoc* analysis showed that control female rats exhibited significantly higher *Nr3c1* mRNA levels in the PFC compared to males (p < 0.01; *p* < 0.0001) while female rats subjected to ESI displayed a significant downregulation of *Nr3c1* mRNA levels, compared to the respective control group (*p* < 0.001). Conversely, in male Wistar rats, ESI did not result in any significant alteration of *Nr3c1* mRNA levels in the PFC (*p* > 0.05; Fig. 1D).

**Fig. 2:**
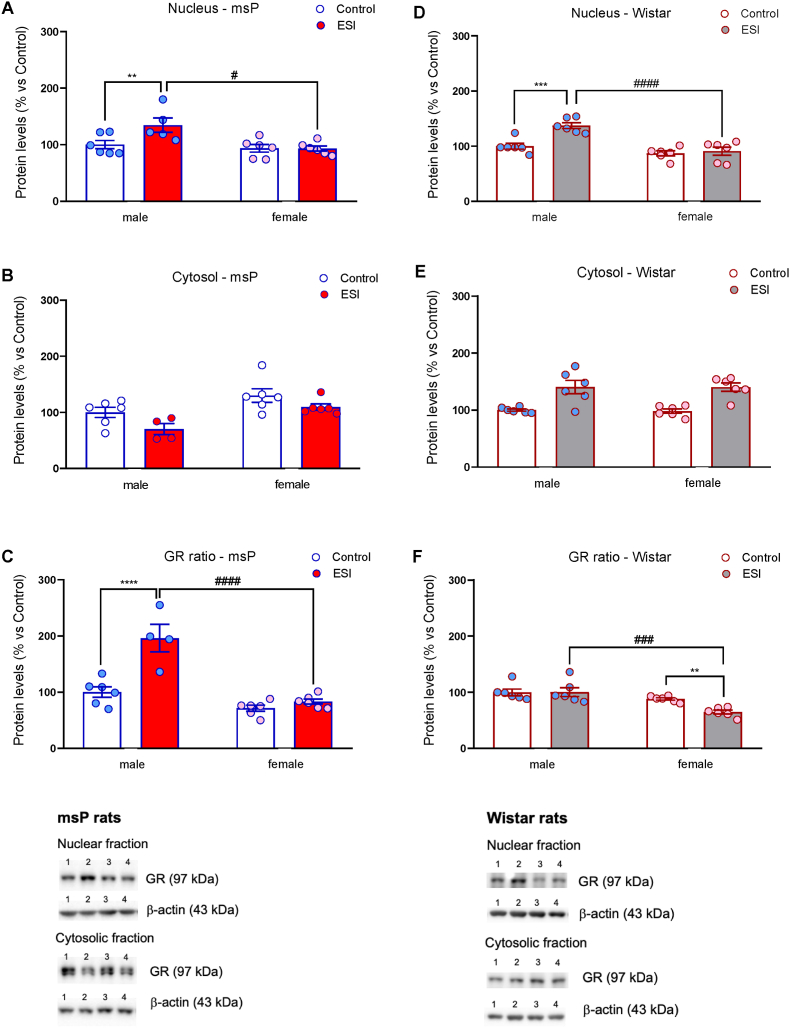
**Effect of early social isolation on GR protein expression in the PFC of male and female msP and Wistar rats.** (**A**, **B**) GR protein expression in the nuclear fraction of PFC in male and female msP (N = 5–6/group) and Wistar (N = 6/group) rats. (**C**, **D**) GR protein expression in the cytosolic fraction of PFC in male and female msP (N = 5–6/group; 1 outlier in male ESI group) and Wistar (N = 6/group) rats. (**E**, **F**) Ratio between nuclear and cytosolic GR protein levels in the PFC in msP (N = 5–6/group; 1 outlier in male ESI group) and Wistar (N = 6/group) rats. Representative cropped Western blot bands for GR (97 kDa) and β-actin (43 kDa) are shown in the lower panel. The data, expressed as % of control male rats, represent the mean ± SEM. **p < 0.01, ***p < 0.001, ****p < 0.0001, #*p <* 0.05, ###*p <* 0.001, ####*p <* 0.0001 two-way ANOVA followed by Newman-Keuls test. Gel image: 1 = control male rats; 2 = ESI male rats; 3 = control female rats; 4 = ESI female rats.

Because no changes in *Nr3c1* mRNA levels were detected in the Amy of msP and Wistar rats, we next measured GR protein levels in nuclear and cytosolic fractions and the nuclear/cytosolic GR ratio as an index of receptor translocation from the cytosol to the nucleus in the PFC only. In the nucleus of both msP (Fig. 2A) and Wistar (Fig. 2B) rats, two-way ANOVA of GR protein levels revealed a significant effect of sex (msP: *F*_1,19_ = 8.78, *p* = 0.008; Wistar: *F*_1,20_ = 26.47, *p* < 0.0001), ESI (msP: *F*_1,19_ = 4.51, *p* = 0.047; Wistar: *F*_1,20_ = 12.63, *p* = 0.002) and a sex × ESI interaction (msP: *F*_1,19_ = 4.81, *p* = 0.041; Wistar: *F*_1,20_ = 8.26, *p* = 0.009). The *post hoc* analysis showed an increased GR expression in ESI-exposed male rats in both msP (*p <* 0.01 vs control male rats; *p <* 0.05 vs ESI female rats) and Wistar (*p <* 0.001 vs control male rats; *p <* 0.0001 vs ESI female rats) strains. Two-way ANOVA of GR protein levels in the cytosol revealed a significant effect of sex (*F*_1,18_ = 12.94, *p* = 0.002), ESI (*F*_1,18_ = 6.82, *p* = 0.02) and no sex × ESI interaction (*F*_1,18_ = 0.21, *p* = 0.647) in msP rats (Fig. 2C), whereas in Wistar rats only ESI condition (*F*_1,20_ = 32.47, *p* < 0.0001) showed a significant effect (Fig. 2D). Two-way ANOVA of nuclear/cytosol GR ratio in both msP (Fig. 2E) and Wistar (Fig. 2F) rats revealed a significant effect of sex (msP: *F*_1,18_ = 43.19, *p* < 0.0001; Wistar: *F*_1,20_ = 19.63, *p* = 0.0003), ESI (msP: *F*_1,18_ = 25.02, *p* < 0.0001; Wistar: *F*_1,20_ = 4.78, *p* = 0.04) and a sex × ESI interaction (msP: *F*_1,18_ = 15.25, *p* = 0.001; Wistar: *F*_1,20_ = 5.04, *p* = 0.04). *Post hoc* comparisons showed that, in msP rats, ESI increased the nucleus/cytosol ratio of GR protein only in the PFC of male rats (*p* < 0.0001 vs control male rats; *p* < 0.0001 vs ESI female rats). In Wistar rats, no changes were observed in male rats (*p* > 0.05 vs control male rats), whereas exposure to the ESI protocol reduced GR trafficking in female rats (*p* < 0.01 vs control female rats; *p* < 0.001 vs ESI male rats).

**Fig. 3:**
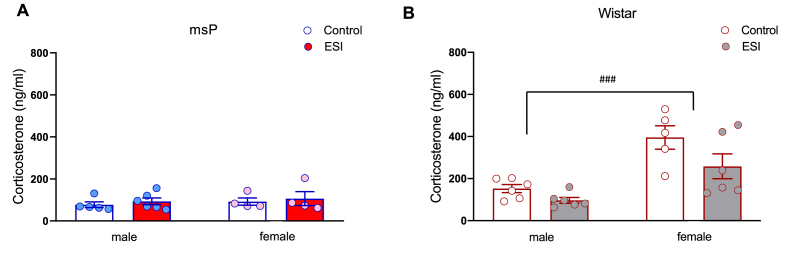
**Effect of early social isolation on plasma corticosterone levels in male and female msP** (**A**) **and Wistar** (**B**) **rats.** Data are expressed in nanogram per milliliter as mean ± SEM. msP rats: N = 5–6/group; 1 outlier in male control group, 1 outlier in female control group, 1 outlier in female ESI group. Wistar rats: N = 6/group; 1 outlier in female control group. The data are expressed as the mean ± SEM. Statistical difference between male and female Wistars: ###*p <* 0.001.

To verify whether the observed dysregulation of the GR system might be associated with altered levels of circulating glucocorticoids, we measured CORT levels in plasma of msP and Wistar rats on PND35. In msP rats, two-way ANOVA revealed no significant effect of sex (*F*_1,15_ = 0.48, *p* = 0.49) or ESI (*F*_1,15_ = 0.57, *p* = 0.46) and no sex × ESI interaction (*F*_1,15_ = 0.003, *p* = 0.96; Fig. 3A). In Wistar rats, two-way ANOVA revealed main effects of sex (*F*_1,19_ = 24.19, *p* < 0.0001) and ESI (*F*_1,19_ = 5.50, *p* = 0.03) but no sex × ESI interaction (*F*_1,19_ = 0.98, *p* = 0.33; Fig. 3B).

### 3.2 EXPERIMENT 2: effect of ESI on FR1 alcohol self-administration

**Fig. 4:**
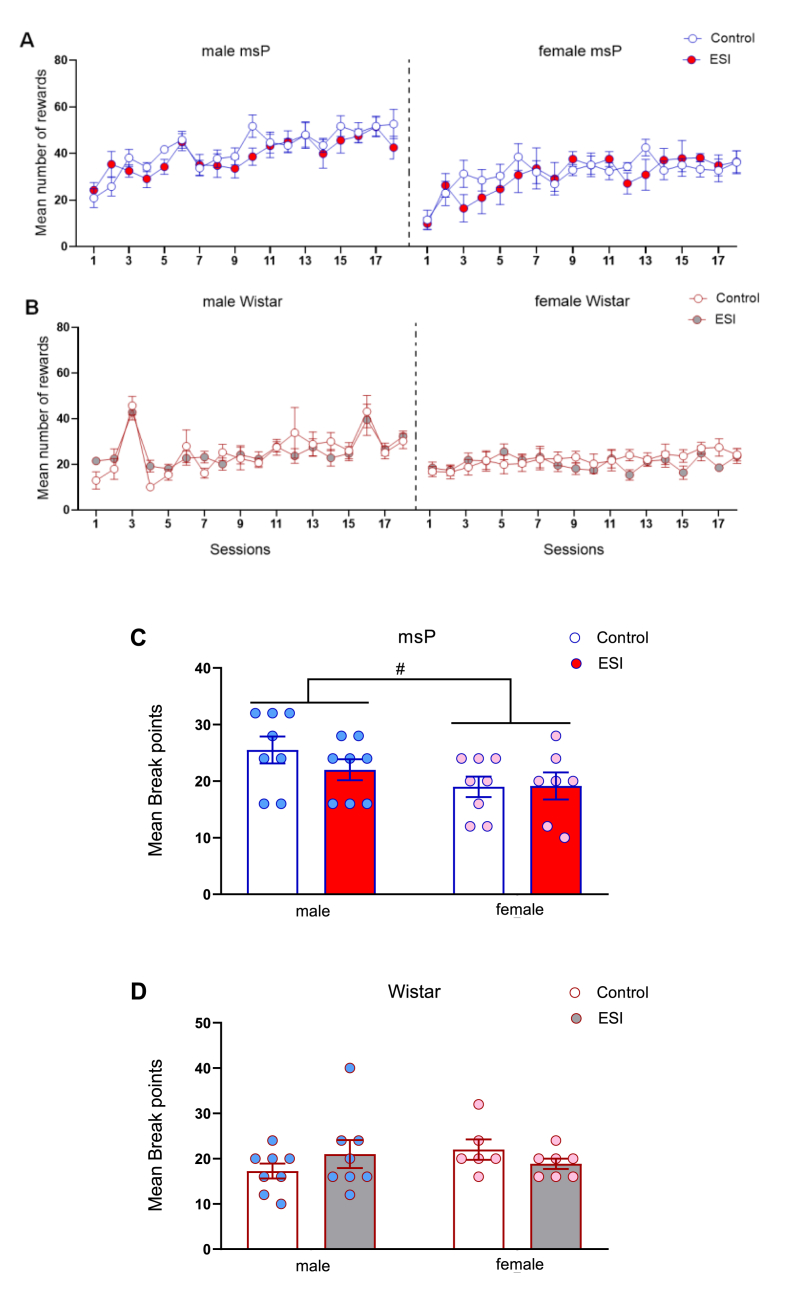
**Effect of early social isolation on alcohol self-administration in male and female msP and Wistar rats.** Acquisition pattern of alcohol self-administration in male and female msP and Wistar rats. Early social isolation did not affect alcohol reinforcement under FR1 schedule in either male (**A, left panel**) or female (**A, right panel**) msP rats or male (**B, left panel**) or female (**B, right panel**) Wistar rats. The motivation for alcohol under the PR contingency in male or female msP rats (**C**) or male or female Wistar rats (**D**) was also not affected. The data are expressed as the mean ± SEM. Main effect of sex: ^#^*p* < 0.05. Where not indicated, differences from controls were not statistically significant.

The effect of ESI on the acquisition of alcohol self-administration was evaluated. In msP rats, the three-way ANOVA revealed overall effects of sessions (*F*_17,486_ = 9.25, *p* < 0.0001) and sex (*F*_1,486_ = 78.50, *p* < 0.0001) but no effect of ESI (*F*_1,486_ = 3.49, *p* = 0.0623) and no interactions. These results reflect a higher number of lever presses in male msP rats throughout training (Fig. 4A, left panel) compared with females (Fig. 4A, right panel). Similarly, the three-way ANOVA of self-administration data in male (Fig. 4B, left panel) and female (Fig. 4B, right panel) Wistar rats revealed significant effects of sessions (*F*_17,450_ = 5.15, *p* < 0.0001) and sex (*F*_1,450_ = 28.89, *p* < 0.0001) and a session × sex interaction (*F*_17,450_ = 3.48, *p* < 0.0001) but no effect of ESI (*F*_1,450_ = 0.56, *p* = 0.45) and no other interactions. Overall, these data suggest that the ESI procedure did not alter the acquisition of alcohol self-administration or responding for alcohol under the FR1 schedule of reinforcement in any of the groups tested, independent of sex and rat strain.

### 3.3 EXPERIMENT 3: effect of ESI on alcohol self-administration on a PR schedule of reinforcement

After the acquisition of a stable baseline of alcohol self-administration under the FR1 contingency, animals were tested in a PR schedule of reinforcement to evaluate their motivation for alcohol. In male and female msP rats, the two-way ANOVA showed a main effect of sex (*F*_1,27_ = 4.89, *p* = 0.035) but no effect of ESI (*F*_1,27_ = 0.63, *p* = 0.43) and no sex × ESI interaction (*F*_1,27_ = 0.74, *p* = 0.40; Fig. 4C). These results suggest that male msP rats, regardless of ESI, exhibited a higher motivation for alcohol. In male and female Wistar rats, the ANOVA revealed no significant effect of sex (*F*_1,25_ = 0.34, *p* = 0.57) or ESI (*F*_1,25_ = 0.018, *p* = 0.89) and no sex × ESI interaction (*F*_1,25_ = 2.37, *p* = 0.14; Fig. 4D).

### 3.4 EXPERIMENT 4: effect of ESI on alcohol self-administration following yohimbine administration

**Fig. 5:**
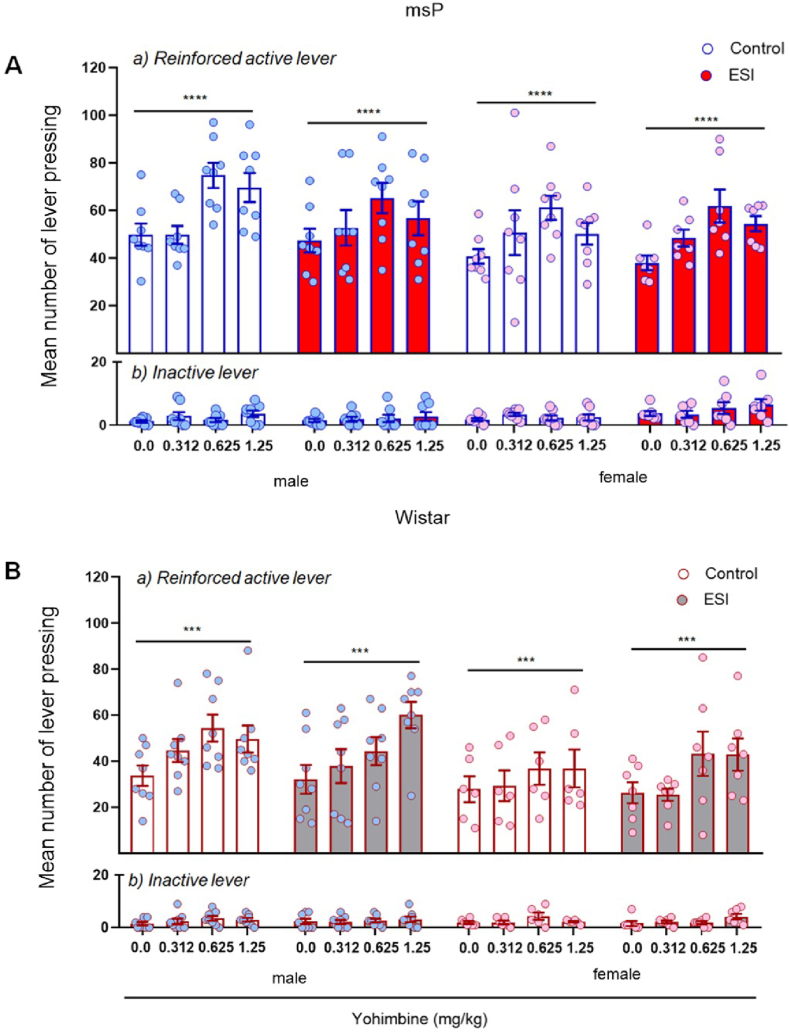
**Effect of early social isolation on alcohol self-administration following yohimbine administration in male and female msP and Wistar rats.** Male and female msP and Wistar rats were treated with the pharmacological stressor yohimbine (0.0, 0.312, 0.625, and 1.25 mg/kg, i.p.) 30 min before the test sessions. Independent of ESI, yohimbine administration increased operant alcohol self-administration in male and female msP rats (**A**) and male and female Wistar rats (**B**). The data are expressed as the mean ± SEM number of (a) reinforced responses (rewards) at the active lever and (b) responses at the inactive lever. Main effect of yohimbine treatment: ***p < 0.001, ***p < 0.001. Where not indicated, differences from controls were not statistically significant.

In msP rats, the three-way ANOVA revealed overall effects of treatment (*F*_3,108_ = 11.02, *p* < 0.0001) and sex (*F*_1,108_ = 7.20, *p* = 0.0084) but no effect of ESI (*F*_1,108_ = 0.94, *p* = 0.34) and no interactions, indicating that yohimbine enhanced alcohol-reinforced lever pressing in both male and females equally, and this effect was not influenced by ESI (Fig. 5A, upper panel). The ANOVA of inactive lever responding showed a significant effect of sex (*F*_1,108_ = 7.67, *p* = 0.0066) and a sex × ESI interaction (*F*_1,108_ = 6.22, *p* = 0.014) but no effect of treatment (*F*_3,108_ = 1.10, *p* = 0.12) or ESI (*F*_1,108_ = 3.84, *p* = 0.05) and no other interactions (Fig. 5A, lower panel).

Similar with msP rats, in Wistar rats, the three-way ANOVA showed significant effects of treatment (*F*_3,100_ = 6.92, *p* = 0.0003) and sex (*F*_1,100_ = 12.27, *p* = 0.0007) but no effect of ESI (*F*_1,100_ = 0.001, *p* = 0.97) and no interactions, indicating that yohimbine increased alcohol self-administration in all experimental groups (Fig. 5B, upper panel). The ANOVA of inactive lever responding showed no significant effect of treatment (*F*_3,100_ = 2.22, *p* = 0.90), sex (*F*_1,100_ = 0.01, *p* = 0.92), or ESI (*F*_1,100_ = 0.07, *p* = 0.79) and no significant interactions (Fig. 5B, lower panel).

### 3.5 EXPERIMENT 5: effect of ESI on yohimbine-induced reinstatement of alcohol seeking

**Fig. 6:**
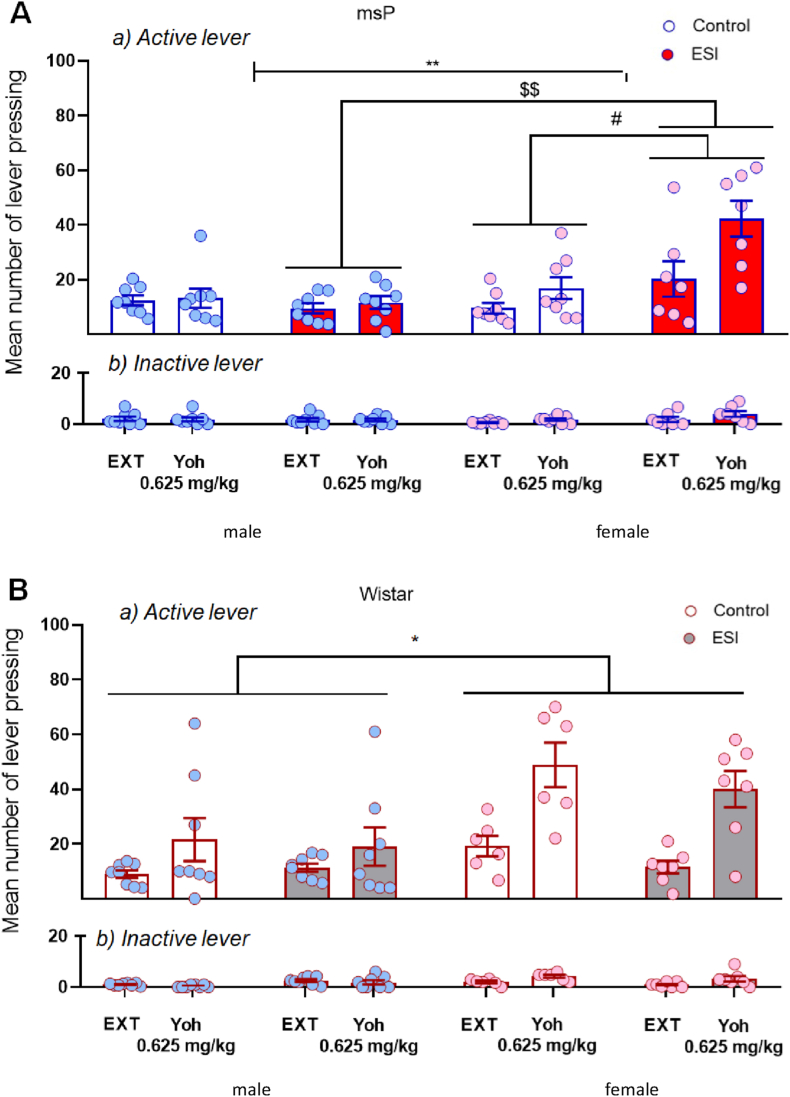
**Effect of early social isolation on yohimbine-induced reinstatement of alcohol seeking in male and female msP and Wistar rats.** Following alcohol self-administration training, male and female msP and Wistar rats were subjected to an extinction phase and then treated with yohimbine (0.625 mg/kg, i.p.). Thirty minutes later, the reinstatement of responding was evaluated. Extinction (EXT): mean number of lever presses during the last 3 days of extinction. Yohimbine administration elicited the significant reinstatement of responding in female msP rats but not in male msP rats. Early social isolation increased the level of reinstatement in female msP rats only (**A**). Similarly, yohimbine reinstated alcohol seeking in female but not male Wistar rats. Early social isolation did not potentiate the effect of yohimbine (**B**). The data are expressed as the mean ± SEM of (a) total responses at the active lever and (b) inactive lever. Sex ⋅ treatment interaction: *p < 0.05, **p < 0.01; treatment ⋅ ESI interaction: #*p* < 0.001; sex ⋅ ESI interaction: $$ *p* < 0.01. Where not indicated, differences from controls were not statistically significant.

Rats that were trained under a FR1 schedule of operant alcohol self-administration were subjected to an extinction phase, during which lever pressing progressively decreased and then was tested for yohimbine-induced reinstatement. During training, the mean number of active lever presses relative to the last 3 days of alcohol self-administration in msP rats were the following: male controls (77.5 ± 6.6), male ESI (68.6 ± 7.8), female controls (45.8 ± 4), female ESI (57.4 ± 6.4). Responding in Wistar rats was the following: male controls (44.5 ± 4), male ESI (42.2 ± 5.4), female controls (43.4 ± 1.2), female ESI (31.4 ± 2.6). During the extinction phase, responding at the active lever progressively decreased. The mean numbers of lever presses during the last 3 days of extinction in msP rats were the following: male controls (12.5 ± 1.8), male ESI (9.5 ± 1.8), female controls (9.5 ± 1.9), female ESI (19.5 ± 6.6). Responding in Wistar rats was the following: male controls (8.9 ± 1.4), male ESI (11.4 ± 1.5), female controls (19.2 ± 3.5), female ESI (11.5 ± 2.3). Following yohimbine administration, in msP rats, the three-way ANOVA showed overall effects of treatment (*F*_1,27_ = 17.69, *p* = 0.0003), sex (*F*_1,27_ = 10.3, *p* = 0.0034), and ESI (*F*_1,27_ = 5.79, *p* = 0.0232), a significant treatment ⋅ sex interaction (*F*_1,27_ = 11.95, *p* = 0.0018), a treatment ⋅ ESI interaction (*F*_1,27_ = 4.371, *p* = 0.00461), a sex ⋅ ESI interaction (*F*_1,27_ = 9.62, *p* = 0.005), but no treatment ⋅ sex ⋅ ESI interaction (*F*_1,27_ = 3.07, *p* = 0.09; Fig. 6A, upper panel). Inactive lever responding was unaffected. The ANOVA revealed no effect of treatment (*F*_1,27_ = 2.16, *p* = 0.15), sex (*F*_1,27_ = 0.18, *p* = 0.67), or ESI (*F*_1,27_ = 1.80, *p* = 0.19) and no interactions (Fig. 6A, lower panel).

In Wistar rats, the ANOVA revealed overall effects of yohimbine treatment (*F*_1,25_ = 29.53, *p* < 0.0001) and sex (*F*_1,25_ = 12.31, *p* = 0.002) but no effect of ESI (*F*_1,25_ = 0.10, *p* = 0.33). The overall ANOVA also revealed a significant treatment ⋅ sex interaction (*F*_1,25_ = 6.87, *p* = 0.015) but no treatment ⋅ ESI interaction (*F*_1,25_ = 0.19, *p* = 0.67), sex ⋅ ESI interaction (*F*_1,25_ = 0.95, *p* = 0.34), or treatment ⋅ sex ⋅ ESI interaction (*F*_1,25_ = 0.07, *p* = 0.79; Fig. 6B, upper panel). Inactive lever presses were negligible and not significantly affected by yohimbine treatment (*F*_1,25_ = 4.01, *p* = 0.06), sex (*F*_1,25_ = 6.71, *p* = 0.02), or ESI (*F*_1,25_ = 0.31, *p* = 0.58), with no treatment ⋅ sex interaction (*F*_1,25_ = 14.26, *p* = 0.001), treatment ⋅ ESI interaction (*F*_1,25_ = 0.003, *p* = 0.95), sex ⋅ ESI interaction (*F*_1,25_ = 7.77, *p* = 0.01), or treatment ⋅ sex ⋅ ESI interaction (*F*_1,25_ = 0.01, *p* = 0.93; Fig. 6B, lower panel).

## 4 Discussion

The present findings demonstrate that repeated ESI experiences have effects on glucocorticoid-related mechanisms in a region-specific, sex-specific, and rat strain-specific manner. These observed changes may indicate varying sensitivity to the impact of yohimbine on alcohol abuse-related behaviors later in life.

Specifically, we found sex differences in *Nr3c1* mRNA levels and GR protein levels in the PFC, which were higher in male msP rats on PND35, whereas no changes were observed in female msP rats or male Wistar rats. Contrary to male msP rats, female Wistar rats subjected to ESI exhibited a significant downregulation of *Nr3c1* mRNA and lower GR levels. Differently from PFC, ESI per se did not affect GR transcript or protein expression in the Amy. Basal plasma CORT levels were unaffected by ESI in msP rats whereas, in female Wistar rats, significantly higher CORT levels were detected compared with males, with ESI decreasing it in both sexes. When rats were subsequently tested for basal alcohol self-administration, the results showed higher alcohol consumption in msP rats compared to Wistars. Drinking was not affected by ESI.

In the present study, we applied ESI during the juvenile period (week 3), during which a large reconfiguration of the neuronal epigenome and extensive synaptogenesis occur (Lister et al., 2013). Furthermore, this developmental time window is characterized by the maturation of functions that are crucial for interactions of rodents with their environment, such as visual, motor, and social abilities (Pellis and Pasztor, 1999; Berardi et al., 2000; Rice and Barone, 2000). Although this developmental period is characterized by significant neuroplasticity, we did not observe major effects of ESI on drinking. Previous studies applied maternal separation during earlier maturation phases (weeks 1 and 2), showing significant changes in the function of multiple brain areas that are involved in stress/reward processing that is often associated with high alcohol drinking in adulthood (Plotsky et al., 2005; Whitaker et al., 2013; Gondré-Lewis et al., 2016). Compared with the ESI protocol that was used herein, maternal separation during early life is likely associated with higher levels of physical stress in pups (e.g., hypothermia and alterations of lactation patterns), which may explain the different effects on drinking. Moreover, in these earlier studies, alcohol consumption was evaluated using home-cage two-bottle choice free drinking, whereas we used operant alcohol self-administration in the present study, which more directly captures the motivation for alcohol compared with its *ingesta* (Moffett et al., 2007; Nylander and Roman, 2013; Palm et al., 2013; García-Gutiérrez et al., 2016; De Almeida Magalhães et al., 2017; Odeon et al., 2017; Amancio-Belmont et al., 2020). Notably, consistent with our findings, Lesscher et al. (2015) reported that social isolation from PND21 to PND42 enhanced two-bottle choice home-cage drinking but did not influence operant responding for alcohol under FR or PR schedules of reinforcement (Lesscher et al., 2015).

Few earlier mouse studies that applied ESI during the third week of life demonstrated an increase in depressive-like behavior or enhanced cocaine-induced conditioned place preference in adulthood, suggesting greater motivation for this psychostimulant (Lo Iacono et al., 2015, 2016, 2017). Based on these data, we would have also expected greater motivation for alcohol, especially in msP rats because they exhibit depressive- and anxiety-like traits that are attenuated by alcohol consumption (Ciccocioppo et al., 1999; Gozzi et al., 2013; Stopponi et al., 2018; Borruto et al., 2021). Contrary to this expectation, we did not observe any effect of ESI on the motivation for alcohol. The different drugs of abuse that were tested (i.e., cocaine *vs*. alcohol) and the fact that previous studies were conducted in mouse pups that were exposed to an additional stressor (i.e., the presence of a resident adult mouse) during the 30-min social isolation session may account for these discrepancies (Lo Iacono et al., 2015, 2016, 2017).

To examine whether stressful stimuli later in life interact with ESI to influence alcohol intake, we also tested the effect of the pharmacological stressor yohimbine on alcohol self-administration and alcohol-seeking behavior. Yohimbine is an α_2_ adrenergic receptor antagonist that increases norepinephrine cell firing (Aghajanian and VanderMaelen, 1982) and enhances norepinephrine release in terminal areas (Abercrombie et al., 1988; Pacak et al., 1992). Yohimbine induces anxiety-like responses in both humans [Holmberg and Gershon (1961); Bremner et al. (1996b) and laboratory animals (Bremner et al., 1996b) and craving in alcohol-dependent patients (Umhau et al., 2011). In the present study, yohimbine increased alcohol-reinforced lever pressing in all experimental groups, regardless of rearing conditions, genotype and sex, thus indicating that ESI does not alter the propensity to drink in response to this pharmacological stressor. A few earlier studies examined the effect of exposure to stressors other than yohimbine on alcohol intake in animals that were subjected to maternal separation during early life, revealing either no difference (Thompson et al., 2020) or an increase in alcohol intake (Roman et al., 2004; Peñasco et al., 2015; García-Gutiérrez et al., 2016).

To our knowledge, no prior studies have examined the consequences of early life stress on later susceptibility to relapse in response to a yohimbine challenge. In the present study, based on the finding that 0.625 mg/kg yohimbine increased alcohol self-administration in both msP and Wistar rats, we tested this dose on the reinstatement of extinguished alcohol seeking. This dose is lower than the dose (1.25 m/kg) that was classically used in previous studies. We chose this dose to better capture potential interactions with ESI (Lê et al., 2005; Marinelli et al., 2007; Ayanwuyi et al., 2013). The results showed that this relatively low dose of yohimbine significantly reinstated alcohol seeking in female but not male rats. In previous studies, the significant reinstatement of alcohol seeking in male rodents was observed following 1.25 mg/kg yohimbine administration (Lê et al., 2005; Marinelli et al., 2007; Ayanwuyi et al., 2013). Therefore, our data suggest that females are more sensitive to yohimbine (Curley et al., 2022). Consistent with this finding, previous studies reported higher yohimbine-induced reinstatement of cocaine seeking in female animals compared with male animals (Anker and Carroll, 2010; Bertholomey et al., 2016). This finding also aligns with clinical work indicating that women who abuse cocaine or alcohol are more likely to relapse in response to stressful events (Fox and Sinha, 2009; Guinle and Sinha, 2020).

Notably, in msP rats, yohimbine elicited significant reinstatement of alcohol seeking only in females that were subjected to ESI. The reason for this difference between female msP and Wistar rats is difficult to explain based on the present molecular data. However, these two rat lines differ significantly in their HPA axis reactivity to ESI. In fact, CORT levels in msP rats are not affected by ESI in both males and females, whereas in Wistar rat females show higher plasma CORT concentrations with ESI reducing CORT levels independently from the sex. Even though we cannot exclude that the hormonal levels in msP rats might have been altered at different time points, these data suggest that the two rat lines differ in their HPA axis reactivity to ESI pointing to Wistar rats as a more responsive strain. Moreover, our results indicate that ESI during the third week of postnatal life affects GR transcription in PFC but not in amygdala. Further, ESI affects GR translocation toward the nucleus in the PFC with differences depending on rat strain and sex. Considering these sex- and rat strain-related readaptations of the glucocorticoid and HPA axis systems it is tempting to speculate that the enhanced susceptibility to yohimbine-induced relapse following ESI, which is observed only in female msP rats, may reflect molecular mechanisms subserving a reduced ability to engage in stress coping behaviors in this group. This hypothesis is corroborated by the evidence that, in ESI Wistar female rats, GR translocation toward the nucleus is reduced, an indication of a less responsive HPA axis, which may be the reason why in this group of Wistars yohimbine induced reinstatement is not enhanced by ESI.

In conclusion, the present results showed that repeated mild social deprivation experiences during the third week of postnatal life led to changes in GR expression in a strain- and sex-dependent manner, with female rats being generally more sensitive to yohimbine-induced alcohol seeking. Moreover, based on the plasma CORT measurements, it is possible to highlight significant hypofunctionality of the HPA axis system in female msP rats compared with Wistar rats because glucocorticoid levels were as low as those in males and were insensitive to prior ESI. Finally data confirm previous findings showing that msP rats display different responsiveness to stress compared to their Wistar counterpart (Natividad et al., 2021; Khom et al., 2023). Our study adds further evidence to previous findings showing that early life adverse events induce sex-dependent effects on alcohol-related behavioral outcomes (Rincón-Cortés, C.A. 2023). However, while studies conducted using either maternal separation or a limited bedding and nesting protocol as early life adverse events found more pronounced effects on alcohol consumption in males than in females, our study reveals a heightened sensitivity of female rats to alcohol seeking evoked by yohimbine compared to males. This suggests that stress modalities and intensity can differentially modulate individual responses depending on sex.

## Funding

This research was funded by NIH/NIAAA grant AA017447 (to MR and RC) AA014351(to FW and RC) and PRIN 2017SXEXT5 (to RC, FF, PR, VT, LF) and by grants from MIUR Progetto Eccellenza 2023–2027 (to FF, VT). FM is recipient of a postdoctoral fellowship from Zardi-Gori foundation.

## CRediT authorship contribution statement

**F. Benvenuti:** Investigation, Writing – original draft. **S. De Carlo:** Investigation. **L. Rullo:** Investigation, Data curation, Formal analysis, Writing - review & editing. **L. Caffino:** Formal analysis, Investigation. **L.M. Losapio:** Investigation, Data curation, Formal analysis. **C. Morosini:** Data curation, Formal analysis. **M. Ubaldi:** Methodology, Supervision. **L. Soverchia:** Data curation, Methodology. **N. Cannella:** Formal analysis, Supervision. **E. Domi:** Formal analysis, Supervision. **S. Candeletti:** Supervision, Formal analysis, Writing – review & editing. **F. Mottarlini:** Formal analysis, Investigation. **L. Fattore:** Conceptualization, Funding acquisition, Writing – review & editing. **P. Romualdi:** Conceptualization, Funding acquisition, Writing - review & editing. **F. Fumagalli:** Conceptualization, Funding acquisition, Methodology, Writing – review & editing, Conceptualization, Funding acquisition, Writing – review & editing. **V. Trezza:** Conceptualization, Funding acquisition, Methodology. **M. Roberto:** Conceptualization, Funding acquisition, Writing – review & editing. **R. Ciccocioppo:** Conceptualization, Funding acquisition, Supervision, Writing – review & editing.

## Declaration of competing interest

The authors declare no competing interests.

## Data Availability

Data will be made available on request.
