# Supplementary material for: Early social isolation differentially affects the glucocorticoid receptor system and alcohol-seeking behavior in male and female Marchigian Sardinian alcohol-preferring rats
Source: Neurobiol Stress. 2023 Dec 7;28:100598. doi: 10.1016/j.ynstr.2023.100598 (PMC10727952; doi:10.1016/j.ynstr.2023.100598)

Supplementary figure S1. Replication of Figure 1 of the main text with the yellow dots representing the oulayers exclude from the statistical analysis

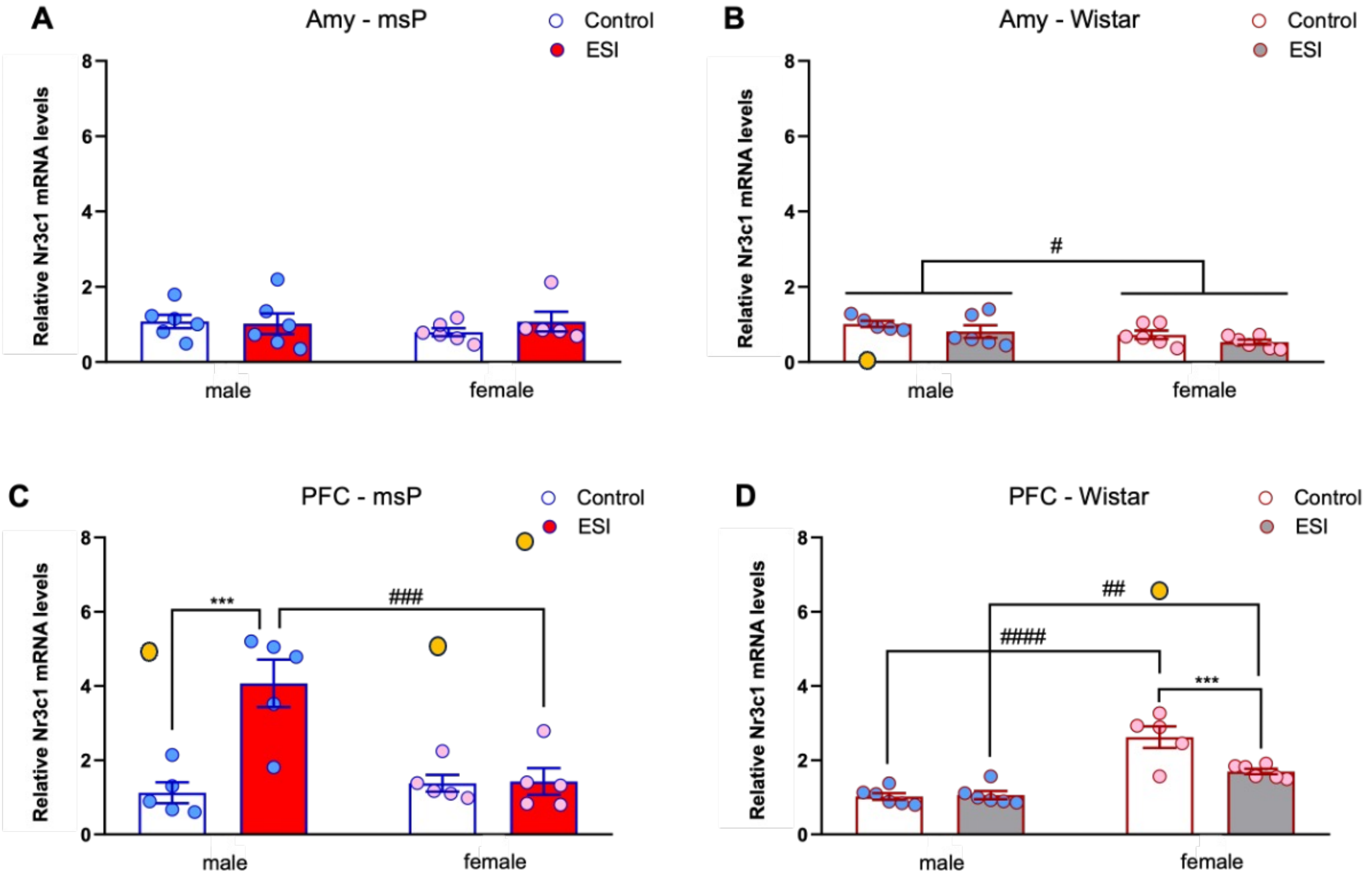

Supplementary figure S1

Supplementary figure S2. Replication of Figure 2 of the main text with the yellow dots representing the oulayers exclude from the statistical analysis

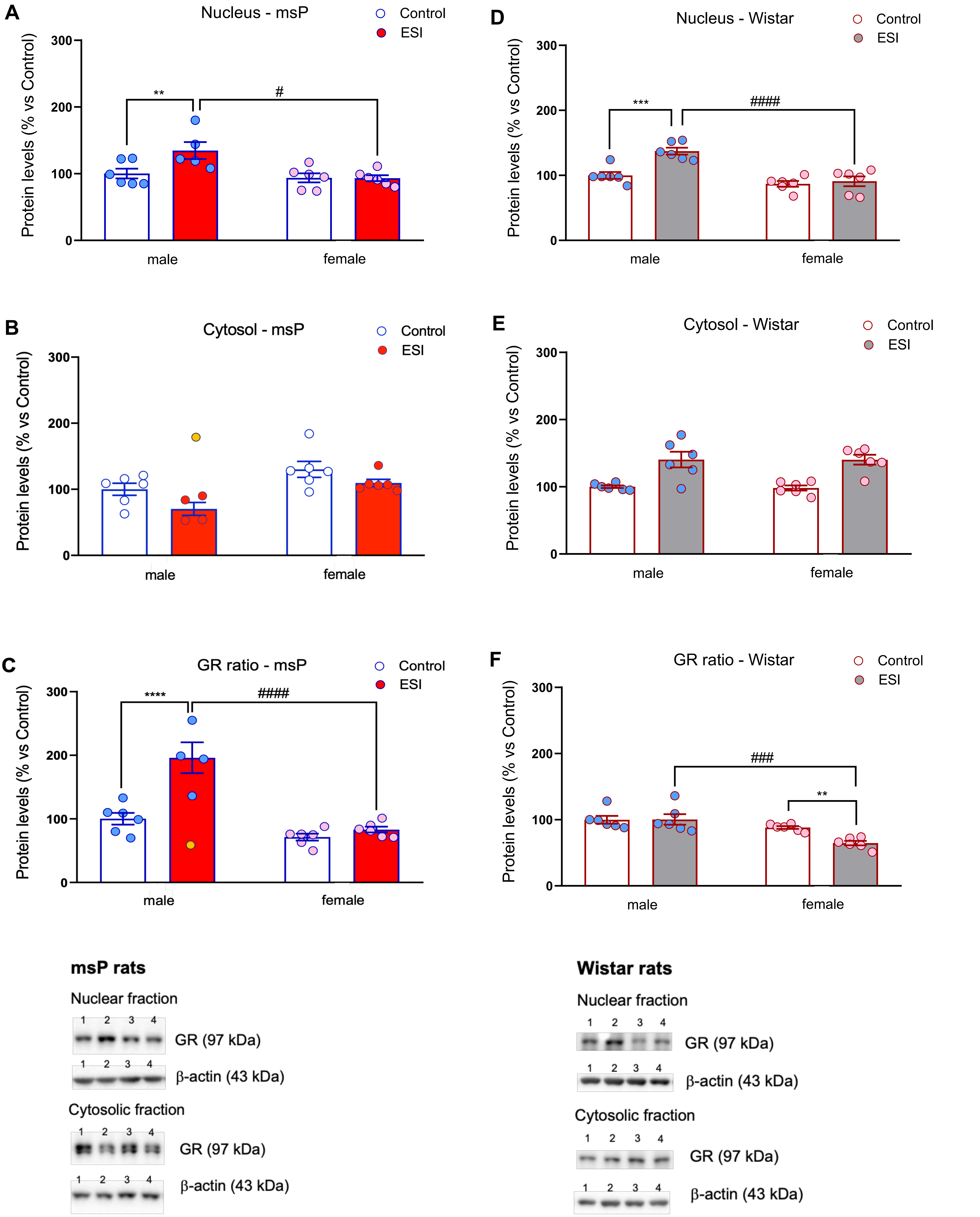

Supplementary figure S3. Example of full-size cropped immunoblots related to the protein expression levels of GR and β-actin measured in the nuclear (**a**) and in the cytosolic fraction (**b**) of the mPFC of control (CTRL) and early social isolated (ESI) male and female msP rats.

1= CTRL male (n=6)

2= ESI male (n=5)

3= CTRL female (n=6)

4= ESI female (n=6)

a

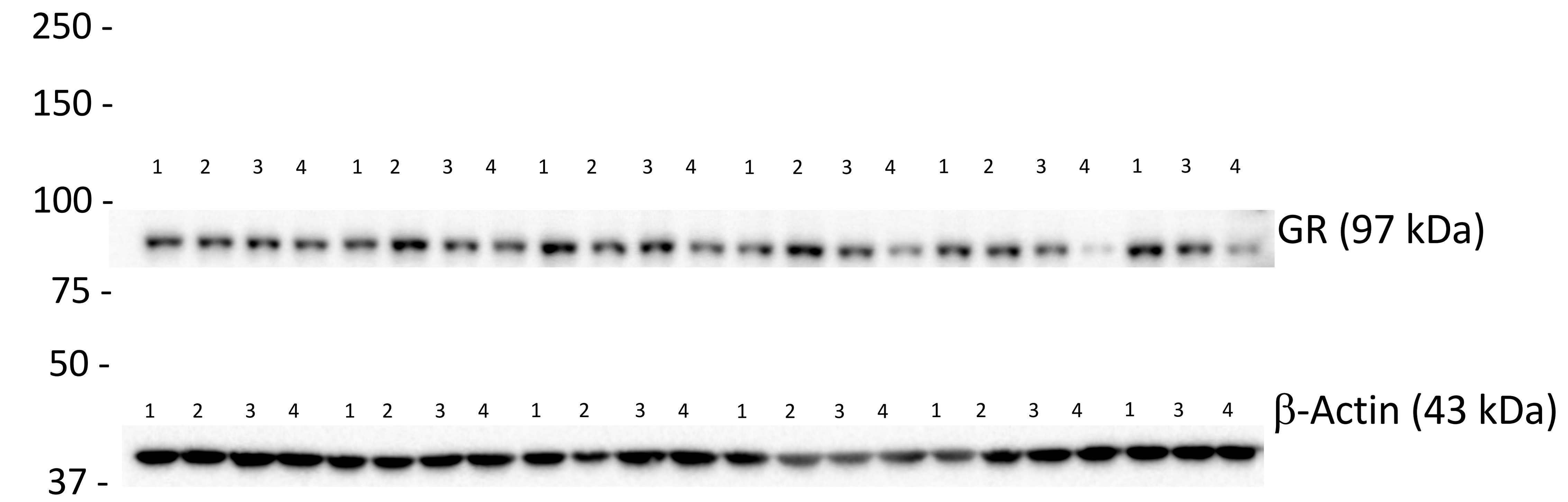

b

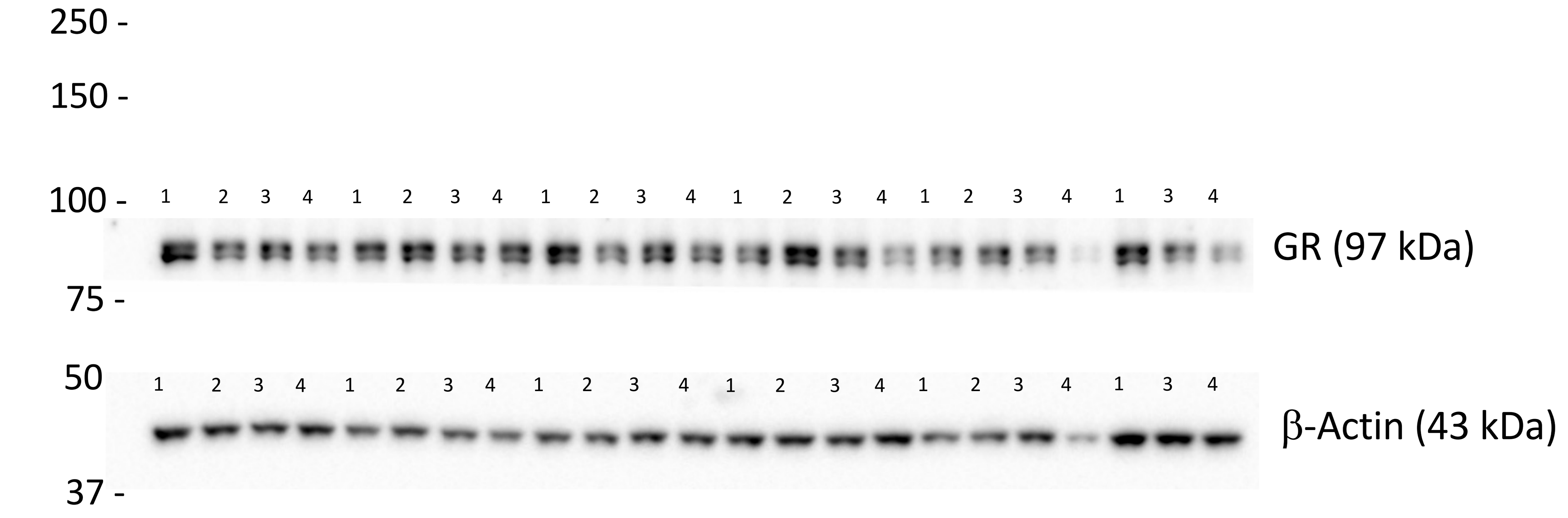

Supplementary figure S4. Example of full-size cropped immunoblots related to the protein expression levels of GR and β-actin measured in the nuclear (**a**) and in the cytosolic fraction (**b**) of the mPFC of control (CTRL) and early social isolated (ESI) male and female Wistar rats.

1= CTRL male (n=6)

2= ESI male (n=6)

3= CTRL female (n=6)

4= ESI female (n=6)

a

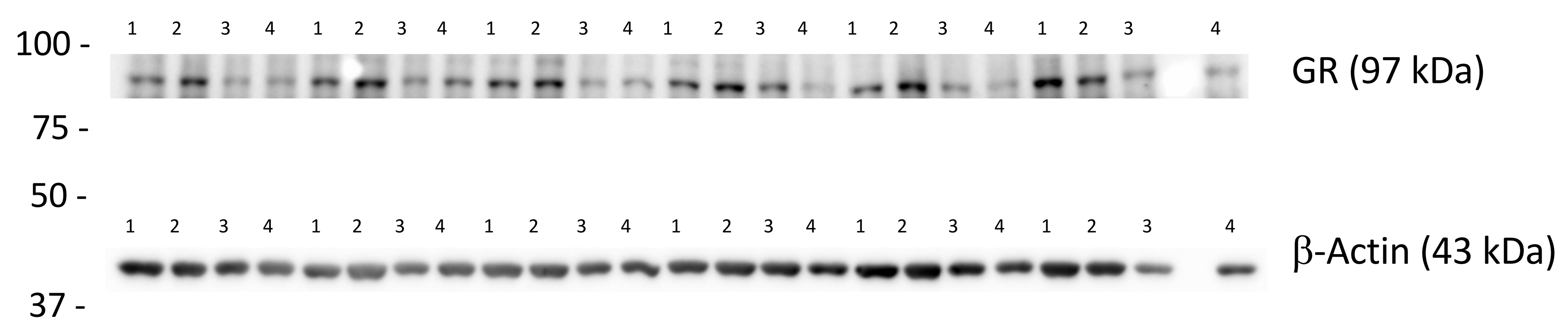

b

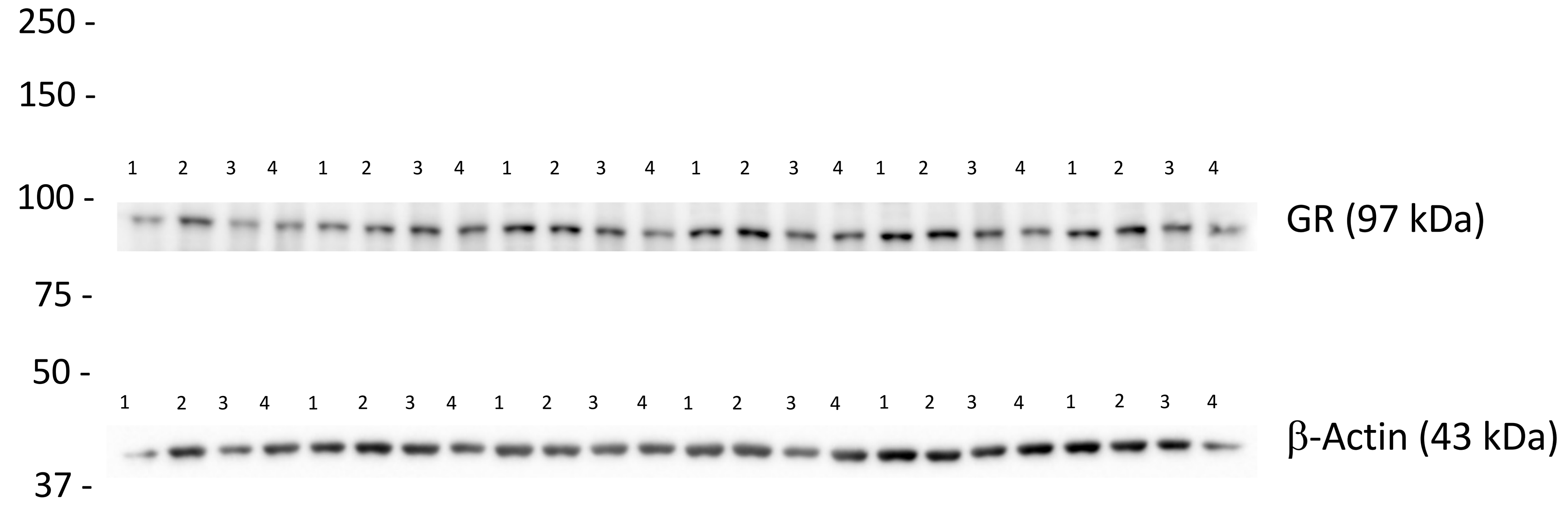

Supplementary figure S5. Replication of Figure 3 of the main text with the yellow dots representing the oulayers exclude from the statistical analysis

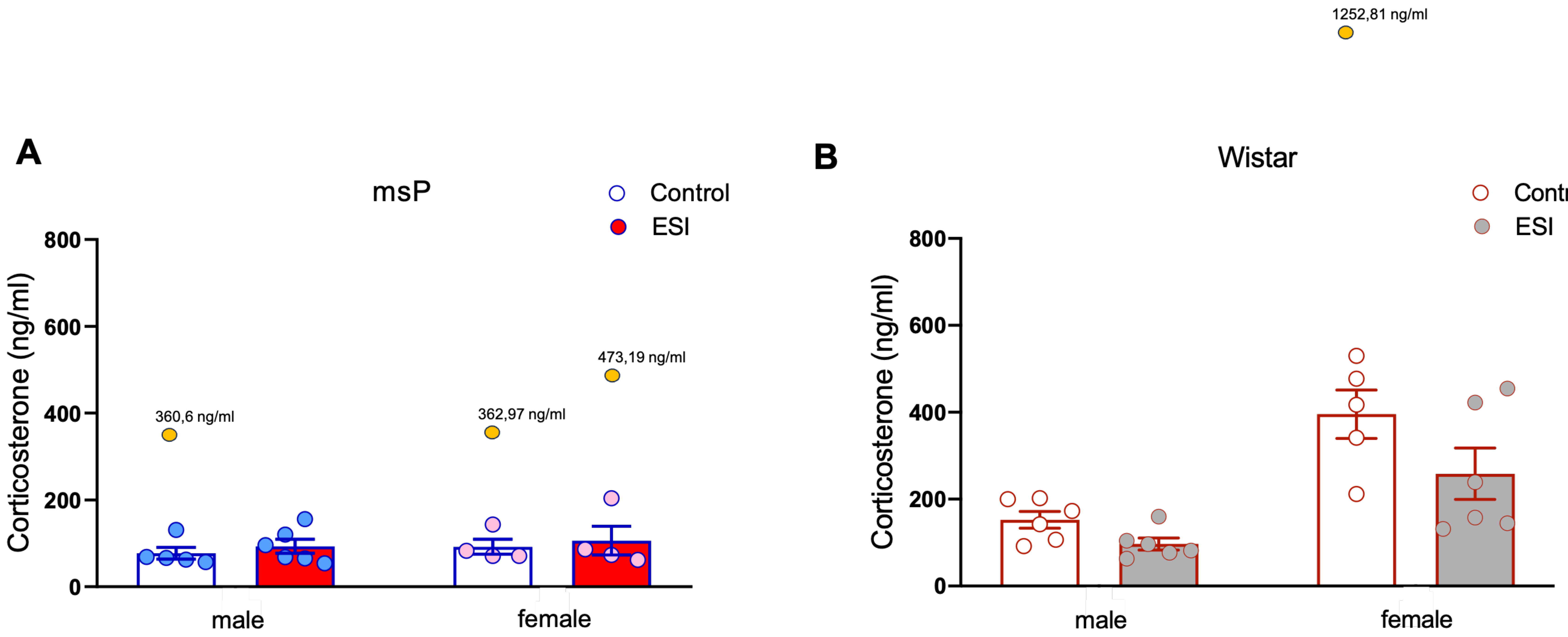

Supplement: Multimedia component 1 [file mmc1.pdf]
